# Glycan cross-feeding activities between bifidobacteria under *in vitro* conditions

**DOI:** 10.3389/fmicb.2015.01030

**Published:** 2015-09-24

**Authors:** Francesca Turroni, Ezgi Özcan, Christian Milani, Leonardo Mancabelli, Alice Viappiani, Douwe van Sinderen, David A. Sela, Marco Ventura

**Affiliations:** ^1^Laboratory of Probiogenomics, Department of Life Sciences, University of Parma, ParmaItaly; ^2^Department of Food Science, University of Massachusetts Amherst, Amherst, MAUSA; ^3^GenProbio s.r.l., ParmaItaly; ^4^Alimentary Pharmabiotic Centre and Department of Microbiology, Bioscience Institute, National University of Ireland, CorkIreland; ^5^Center for Microbiome Research, University of Massachusetts Medical School, Worcester, MAUSA

**Keywords:** microbiota, microbe–microbe interactions, RNAseq, transcriptomics

## Abstract

Bifidobacteria colonize the gut of various mammals, including humans, where they may metabolize complex, diet-, and host-derived carbohydrates. The glycan-associated metabolic features encoded by bifidobacteria are believed to be strongly influenced by cross-feeding activities due to the co-existence of strains with different glycan-degrading properties. In this study, we observed an enhanced growth yield of *Bifidobacterium bifidum* PRL2010 when co-cultivated with *Bifidobacterium breve* 12L, *Bifidobacterium adolescentis* 22L, or *Bifidobacterium thermophilum* JCM1207. This enhanced growth phenomenon was confirmed by whole genome transcriptome analyses, which revealed co-cultivation-associated transcriptional induction of PRL2010 genes involved in carbohydrate metabolism, such as those encoding for carbohydrate transporters and associated energy production, and genes required for translation, ribosomal structure, and biogenesis, thus supporting the idea that co-cultivation of certain bifidobacterial strains with *B. bifidum* PRL2010 causes enhanced metabolic activity, and consequently increased lactate and/or acetate production. Overall, these data suggest that PRL2010 cells benefit from the presence of other bifidobacterial strains.

## Introduction

Bifidobacteria are key gut commensals of human beings, reaching a high-relative abundance when their host is an infant (Ventura et al., 2012; Turroni et al., 2014b). These bacteria have a saccharolytic lifestyle and their metabolism is consequently directed toward the utilization of carbohydrates that are naturally occurring in their ecological niches, which not only include dietary glycans, such as resistant-starch and xylan, but also host-derived glycans, i.e., mucin and human milk oligosaccharides (Turroni et al., 2010, 2011b; Duranti et al., 2014, 2015). In order to access these glycans, bifidobacteria have evolved an enzymatic repertoire of extracellular glycosyl hydrolases (GH) that catalyze the breakdown of such polysaccharides, with the production of mono, di/trisaccharides that are then imported into the cell through the action of specific carriers (Turroni et al., 2012). In addition, other species such as *Bifidobacterium longum* ssp. *infantis* possess apparently unique molecular mechanisms to capture intact oligosaccharides to be further processed intracellularly (Sela, 2011). It should, however, be considered that in their ecological niche bifidobacterial populations may interact with different strains/species which may lead to competition for or co-operative sharing of nutrients.

Biotic interactions between bacteria can either positively or negatively influence the fitness of the affected organisms (Pande et al., 2015). Several of these interactions rely on either the active or passive release of chemical molecules into the environment (Phelan et al., 2012; Morris et al., 2013). In this context, interspecies cross-feeding has been observed for *Bifidobacterium bifidum* PRL2010 and *Bifidobacterium breve* UCC2003 cells when cultivated on sialyllactose as the unique carbon source (Egan et al., 2014a,b). Moreover, previous studies have demonstrated metabolic cross-feeding between *Bifidobacterium adolescentis* and lactate-utilizing, butyrate-producing *Firmicutes* bacteria related to *Eubacterium hallii* and *Anaerostipes caccae* (Belenguer et al., 2006). This is significant and relevant to host health as butyrate is widely regarded as a beneficial short-chain fatty acid produced by elements of the microbiota. Furthermore, bacterial cross-feeding opportunities as facilitated by members of the colonic microbiota have been considered to be pivotal for carbohydrate turn-over in this ecological niche (De Vuyst and Leroy, 2011).

In recent years, extensive scientific efforts have been made to decode bifidobacterial genome sequences, which are part of a novel discipline called probiogenomics, directed to understand the genetics sustaining the adaptation of these bacteria to the intestine (Ventura et al., 2009; Milani et al., 2014). In this context, the genome sequence of *B. bifidum* PRL2010, an infant gut isolate, exhibits several genetic adaptations to the human gut and for this reason is employed as a model strain to investigate the biology of infant-associated bifidobacteria (Turroni et al., 2010, 2013; Serafini et al., 2014). The ability of PRL2010 to utilize host-derived glycans such as mucins and human milk oligosaccharides, and the capacity to produce pilus-like structures to facilitate gut colonization and immuno modulation, are clear examples of such genetic adaptations (Turroni et al., 2010, 2013; Serafini et al., 2014).

Here, we investigate possible cross-feeding activities of simple bifidobacterial communities under *in vitro* conditions, targeting specific complex, diet-associated carbohydrates. Such cross-feeding activities were investigated employing transcriptome analysis of bifidobacterial communities by means of RNAseq as well as by assessment of the metabolic profiles of these bifidobacterial consortia. The main findings of the current study are that *B. bifidum* PRL2010 does not utilize starch or xylan, unless co-cultured with a strain that produces extracellular glycoside hydrolases that can degrade these substrates. These findings provide evidence of mutalisitic cross-feeding between certain bifidobacterial strains when co-cultured in media containing starch or xylan.

## Materials and Methods

### Growth Conditions

*Bifidobacterium bifidum* PRL2010 (Turroni et al., 2010) on its own, or in combination with *B. breve* 12L. (Bottacini et al., 2014), *B. adolescentis* 22L (Duranti et al., 2014), or *Bifidobacterium thermophilum* JCM1207 were cultivated in an anaerobic atmosphere (2.99% H_2_, 17.01% CO_2_, and 80% N_2_) in a chamber (Concept 400; Ruskin) at 37°C for 24 h in de Man–Rogosa–Sharpe (MRS; Scharlau Chemie, Barcelona, Spain) medium, supplemented with 0.05% (wt/vol) L-cysteine hydrochloride.

### Co-Cultivation

Viable cells of each of the following strains: *B. bifidum* PRL2010, *B. breve* 12L, *B. adolescentis* 22L, or *B. thermophilum* JCM1207, or these strains in co-cultivation with *B. bifidum* PRL2010 was inoculated in 6 ml of MRS (without any carbohydrate; Scharlau Chemie, Barcelona, Spain) supplemented with 1% of either RS2-resistant starch or xylan (Poly(β-D-xylopyranose[1→4]; Sigma–Aldrich) as the sole carbon source in triplicates. Cell suspensions were mixed and incubated at 37°C for 24 h under anaerobic conditions. Bacterial cell cultivations were performed in triplicate (biological replicates).

Bacterial strain enumerations at the beginning and at the end of a given growth experiment were determined by quantitative real-time PCR (qRT-PCR).

### Evaluation of PRL2010 Cell Numbers in Co-Cultivation Trials

Possible enhancement or reduction of PRL2010 growth as a consequence of co-cultivation with other bacteria was monitored by qRT-PCR at the begin as well as at the end of the growth experiments. The amounts of cells for each of the strain used at the begin of the growth evaluation trials determined by qRT-PCR assays are shown in Supplementary Figure S1. qRT-PCR experiments were based on strain-specific primers targeting genes present in single copy within the genomes of PRL2010 (BBPR_0282) 12L (B12L_0105), 22L (BADO_1546), and JCM1207 (BTHER_1915). The copy-number of a gene, and the deduced cell number (since the genes targeted were in single copy per genome) of a given strain used in the co-cultivation experiments was evaluated by comparing the cycle threshold (Ct) values obtained with those from a standard curve. Standard curves were calculated from serial dilutions of a culture with a known cell number (as determined by viable count assessment) for each bacterial strain vs. Ct produced for each target gene. In the case of PRL2010 we used the following primer couple: BBPR_0282-UNI (5′-GCGAACAATGATGGCACCTA-3′) and BBPR_282-REV (5′-GTCGAACACCACGACGATGT-3′). In the case of *B. breve* 12L, we used 12L-UNI (5′-CGAAGTTCCAGTTCACCAT-3′) and 12L-REV (5′-GTTCTTGGCGTTCCAGATGT-3′); for *B. adolescentis* 22L we employed the PCR primers 22L-UNI (5′-GACCAAGCCAACCAGTTCAT-3′) and 22L-REV (5′-TTGGTGGCCTTGTAGTAGCC-3′); and for *B. thermophilum* JCM1207 the following PCR primers BTHERfw (5′-TTACACGCATCCCAATACGC-3′) and BTHERrv (5′-CGTGAAGTATGGATGGTCGC-3′). These primers target genes of the sortase-dependent pilus loci identified in the genomes of these microorganisms (Turroni et al., 2014a).

### Metabolic Profiling

For quantitative determination of metabolites produced by bifidobacterial fermentation of starch and xylan, cell-free supernatants were analyzed using Agilent 1260 Infinity HPLC system equipped with Wyatt Optilab T-rEX Refractive Index detector. Separation was carried out using a Shodex Sugar SH1011 column (8.0 mm ID × 300 mm) at 60°C with the detector temperature maintained at 30°C. The mobile phase was prepared in 20 mM H_2_SO_4_ and run at a flow rate of 0.6 mL/min for 30 min. Injection volume was set at 10 μL and speed of draw and eject was set to 100 μL/min. External sugar standards (glucose, maltose, xylose, and maltotriose) and organic acid (acetic acid and lactic acid) standards were purchased from Sigma–Aldrich Co. (USA). Concentrations of individual sugars and organic acids in samples were calculated from calibration curves drawn from external standards for five different concentrations (0.5, 1, 5, 10, and 20 mg/L). Each metabolic profiling experiment was carried out in triplicate (three measurements were performed for each replicate). Glucose and maltose consumption was calculated by subtracting the values at 24 h from time zero. Since the concentration of metabolites produced is dependent upon the cell density at the end of fermentation (i.e., more cells would yield more endproducts), raw metabolite values were normalized by cell density to correct for differences in biomass.

### RNA Extraction and Purification

Total RNA was isolated using a previously described method (Turroni et al., 2011a). Briefly, cell pellets/tissue materials were resuspended in 1 mL of QUIAZOL (Qiagen, UK) and placed in a tube containing 0.8 g of glass beads (diameter, 106 μm; Sigma). Cells were lysed by shaking the mix on a BioSpec homogenizer at 4°C for 2 min (maximum setting). The mixture was then centrifuged at 12,000 rpm for 15 min, and the upper RNA-containing phase was recovered. The RNA sample was further purified by phenol extraction and ethanol precipitation according to an established method (Sambrook and Russel, 2001). RNA quality was checked by analyzing the integrity of rRNA molecules by a Tape Station (Agilent Technologies).

### RNAseq with Ion Torrent Personal Genome Machine (PGM)

One hundred nanogram of total RNA was used as the starting input for RNA-Seq library preparation. Briefly, 100 ng of total RNA was treated with MICROB*Express*^TM^ Bacterial RNA Enrichment Kit (Ambion) to remove rRNA according to the supplier’s instructions. The efficacy of rRNA depletion was checked by a Tape Station (Agilent Technologies), after which rRNA-depleted RNA samples were fragmented using RNaseIII (Life Technologies, USA) followed by size evaluation using a Tape Station (Agilent Technologies). Whole transcriptome libraries were constructed using the Ion Total-RNA Seq Kit v2 (Life Technologies, USA). Barcoded libraries were quantified by qRT-PCR and each library template was amplified on Ion Sphere Particles using Ion One Touch 200 Template Kit v2 (Life Technologies, USA). The samples were loaded on 316 Chips and sequenced by means of a PGM instrument (Life Technologies, USA). Sequencing reads were depleted of adapter sequences and quality filtered (with overall quality, quality window, and length filters) using a custom script implying fastq-mcf^1^ (settings: –qual-mean=25, -w 5 –q 20 –l 100). The resulting processed sequences were aligned to the reference genomes through BWA (Li and Durbin, 2009) with mismatch and gap penalty increased to six and eight, respectively, in order to avoid cross-mapping during analysis of co-cultivation samples. Reference genomes used for RNASeq bioinformatics analyses are deposited under the following accession numbers: CP006711 (*B. breve* 12L), CP007443 (*B. adolescentis* 22L), CP001840 (*B. bifidum* PRL2010), and GCA_000741495.1 (*B. thermophilum* JCM1207). Counting of the number of reads that correspond to annotated open reading frames (ORFs) was performed using HTSeq^2^ and analysis of the count data was performed using the R package DESeq (Anders and Huber, 2010).

### Statistical Analysis

Statistical significance between means was analyzed using the unpaired Student’s *t*-test . Values are expressed as the means ± SE of the mean of a given experiment performed in triplicate.

### Sequence Accession Numbers

All RNAseq raw data from this study were deposited in the SRA database under the accession number SRP058697 (BioProject accession number PRJNA284883).

## Results and Discussion

### Evaluation of the Growth Performances on Dietary Polysaccharide

**FIGURE 1:**
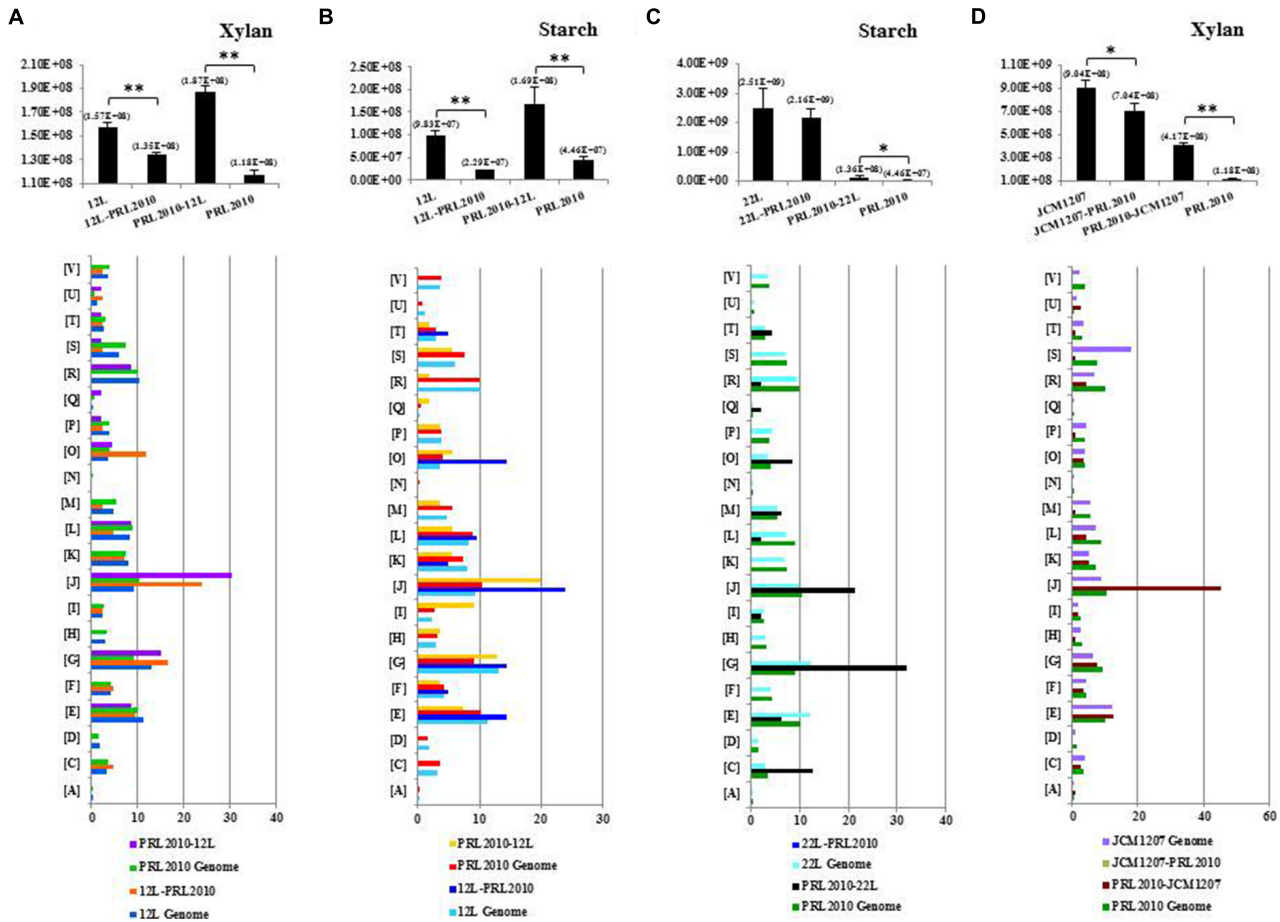
**Transcriptome analyses of co-cultivated bifidobacterial strains.** Only bi-associations showing a cross-feeding behavior between bifidobacterial strains are represented. **(A–D)** The cell number evaluation of *Bifidobacterium breve* 12L, *Bifidobacterium bifidum* PRL2010, *Bifidobacterium adolescentis* 22L, and *Bifidobacterium thermophilum* JCM1207 strains in mono- and co-cultivation on the glycans-based medium indicated above each panel by qRT-PCR. The bar plot placed below represented the functional annotation of expressed genes of bi-association according to their cluster orthologous gene (COG) categories. Results of qRT-PCR are represented in pillars in which the *y*-axis represents the genome copy number/ml of bacterial culture and *x*-axis showed the name of the strains involved in mono- and bi-associations. The value in parenthesis above each pillar represents the average cell numbers for that condition. The color of each COG family is indicated in the figure. Each COG family is identified by one-letter abbreviations: A, RNA processing and modification; B, chromatin structure and dynamics; C, energy production and conversion; D, cell cycle control and mitosis; E, amino acid metabolism and transport; F, nucleotide metabolism and transport; G, carbohydrate metabolism and transport; H, coenzyme metabolism; I, lipid metabolism; J, translation; K, transcription; L, replication and repair; M, cell wall/membrane/envelop biogenesis; N, cell motility; O, post-translational modification, protein turnover, chaperone functions; P, inorganic ion transport and metabolism; Q, secondary structure; T, signal transduction; U, intracellular trafficking and secretion; Y, nuclear structure; Z, cytoskeleton; R, general functional prediction only; S, function unknown. The percentage was calculated as the percentage of transcribed genes belonging to the indicated COG category with respect to all transcribed genes. Asterisks indicate that the presented data display a significant, either ^∗^*p* < 0.05 or ^∗∗^*p* < 0.01, deviation from the obtained values of the mono-association.

Growth capabilities of *B. bifidum* PRL2010, *B. adolescentis* 22L, *B. breve* 12L, and *B. thermophilum* JCM1207 cultivated on their own (mono-association) on MRS supplemented with starch or xylan as the sole carbon source were evaluated (**Figure 1**; Supplementary Figure S1) and compared to those achieved when these strains were co-cultivated in pairs (bi-associations) on identical substrates. In addition, cultivation experiments involving all the strains used in this study were also performed on MRS without carbon source, revealing, as expected, the absence of any sign of growth. These carbohydrates were selected in order to represent carbon sources that may occur in the human gut as they are derived from a plant-derived, glycan-based diet (Chassard and Lacroix, 2013). Notably, of all the possible strain pair combinations, only those bi-associations that included PRL2010 cells displayed significant differences in growth with respect to the respective mono-associations (**Figure 1**; Supplementary Figure S1). As expected, PRL2010 cells did not exhibit any significant growth on starch or xylan when grown in a mono-culture (Turroni et al., 2012). However, when this strain was co-cultivated with 22L or 12L cells on a MRS-supplemented with starch, the number of cells increased (about three- and fourfold, respectively, *p* < 0.005) compared to the situation of mono-association, respectively (**Figure 1**). In contrast, 12L appears to significant decrease (compared to mono-association; fourfold, *p* < 0.001) during growth on starch when co-cultivated with the presence of PRL2010 cells (**Figure 1**). Conversely, *B. bifidum* PRL2010 did not appear to influence the growth yields (changes less than twofold) of 22L cells on starch, or that of 12L when cultivated on xylan, as displayed by unchanged growth yields of bi-association compared to mono-association of these strains (**Figure 1**). Another interesting increase in deduced PRL2010 cell numbers was observed for the bi-association of PRL2010 with *B. thermophilum* JCM1207 (threefold, *p* < 0.001), when grown on MRS supplemented with xylan (**Figure 1**). In contrast, JCM1207 cells exhibit a modest reduction in growth ability (relative to mono-association), when co-cultivated with PRL2010 (**Figure 1**; Supplementary Figure S1). Overall, the concomitant presence of two different bifidobacterial strains was shown in some cases to increase the cell numbers of one partner when cultivated on complex carbohydrates, thus suggesting cross-feeding abilities of bifidobacterial strains.

### Assessing the Metabolic Profile of Bifidobacteria

**FIGURE 2:**
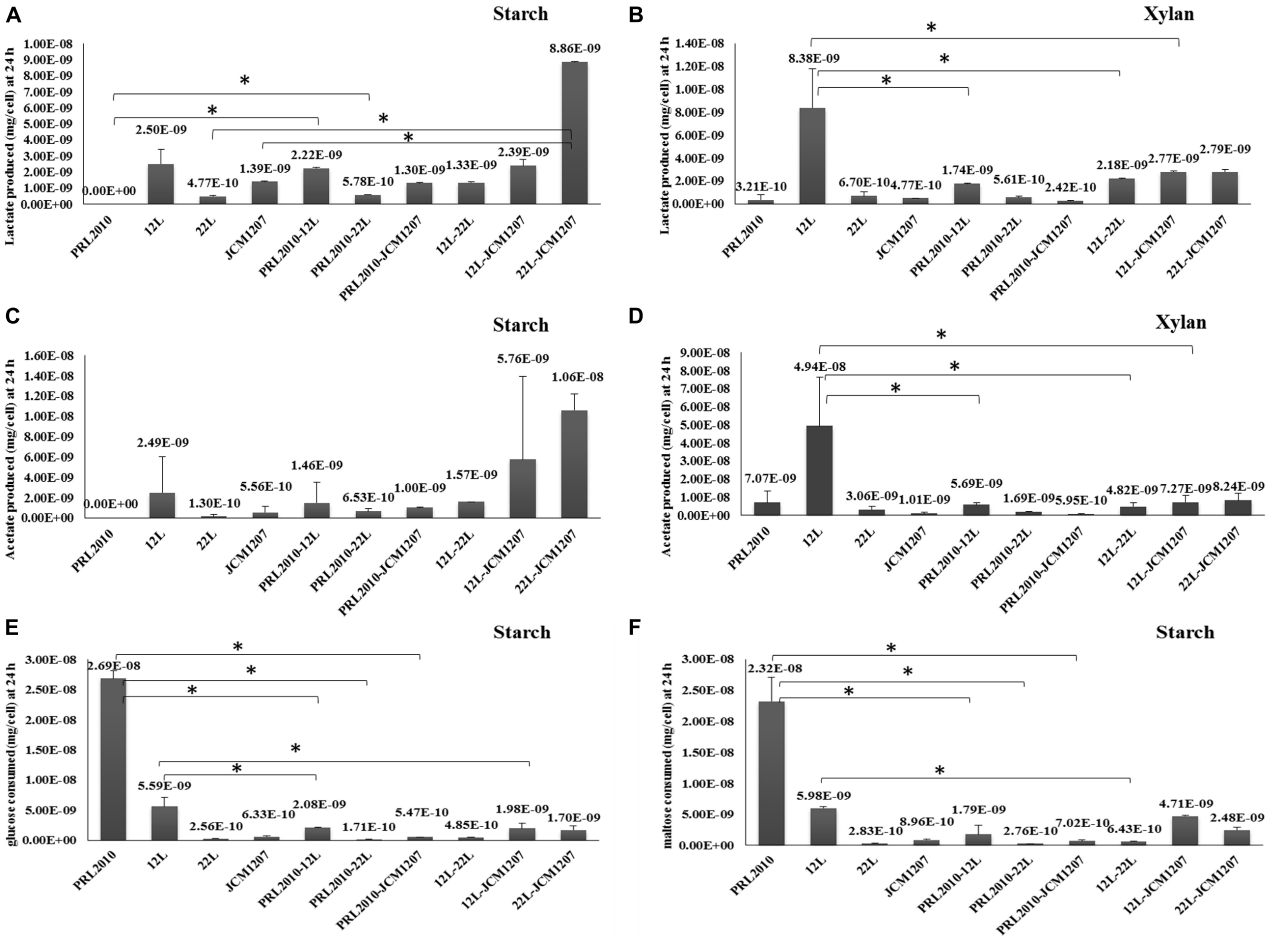
**Metabolic profiling of co-cultivated bifidobacteria. (A,B)** The evaluation of the lactate production of *B. bifidum* PRL2010, *B. breve* 12L, *B. adolescentis* 22L, and *B. thermophilum* JCM1207 strains in mono- and co-cultivation on starch-based and xylan-based medium at 24 h by HPLC, respectively. Values are expressed as mean ± SD mg per cell. **(C,D)** The evaluation of the acetate production of *B. bifidum* PRL2010, *B. breve* 12L, *B. adolescentis* 22L, and *B. thermophilum* JCM1207 strains in mono- and co-cultivation on starch-based and xylan-based medium at 24 h by HPLC, respectively. Values are expressed as mean ± SD mg per cell. **(E)** The evaluation of glucose consumption of *B. bifidum* PRL2010, *B. breve* 12L, *B. adolescentis* 22L, and *B. thermophilum* JCM1207 strains in mono- and co-cultivation on starch-based medium at 24 h by HPLC. Values are expressed as mean ± SD mg per cell. **(F)** The evaluation of maltose consumption of *B. bifidum* PRL2010, *B. breve* 12L, *B. adolescentis* 22L, and *B. thermophilum* JCM1207 strains in mono- and co-cultivation on starch-based medium at 24 h by HPLC, respectively. Values are expressed as mean ± SD mg per cell. Asterisks indicate that the presented data display a significant difference (*p* < 0.05) with respect to those obtained for the mono association. The value in parenthesis above each pillar represents the mean ± SD mg per cell for that condition.

In order to investigate if the co-occurrence of two strains influences bifidobacterial metabolism, we evaluated the production of the metabolic endproducts acetate and lactate, along with the depletion of various sugars from the culture supernatant (i.e., glucose and maltose). The results of this metabolic comparison between mono-associations and bi-associations (as collected from three independent experiments) are depicted in **Figure 2**. Bifidobacteria produce acetate and lactate as a result of their saccharoclastic fermentative metabolism through the so-called bifid shunt (Sela et al., 2008; Pokusaeva et al., 2011). Interestingly, lactate production from starch and xylan fermentation showed significant differences between bifidobacterial species. Whereas acetate production from xylan fermentation varied significantly between certain species in mono-associations (PRL2010 vs. 12L, 12L vs. 22L, and 12L vs. JCM1207), and between 12L and all 12L co-cultivations, its production from starch did not show significant variation between species or bi-associations (**Figure 2C**). Since *B. bifidum* PRL2010 did not exhibit significant growth utilizing starch as a sole carbohydrate, acetate, and lactate production was not observed 24 h into the fermentation. Although growth of PRL2010 was enhanced when co-cultivated with 12L, 22L, or JCM1207 in a medium containing xylan as the sole carbon source, it did not appear to influence acetate and lactate production compared to the respective mono-associations (**Figure 2B**). Interestingly, lactate production during co-cultivation of 22L and JCM1207 in starch increased compared to the respective mono-associations (**Figure 2A**). This may be indicative of bacterial proto-cooperation when grown on starch, as the same relationship was not observed with xylan. *B. breve* 12L cells produced significantly more lactate and acetate while fermenting xylan in pure culture as compared to its bi-association with other species (**Figures 2B,D**). This suggests that 12L energy metabolism may be inhibited by the presence of other bifidobacteria. Interestingly, 12L did not produce biomass when grown in axenic culture, suggesting metabolic flux in the absence of cellular growth. Glucose and maltose depletion during starch fermentation varied among species. *B. breve* 12L cells consumed more glucose alone than when co-cultivated with PRL2010, JCM1207, and 22L cells in starch fermentation (**Figures 2E,F**). However, this did not coincide with a significant decrease in lactate production (**Figure 2A**). While fermenting starch individually, 22L and JCM1207 consumed similar amounts of glucose (2.56E-10 and 6.33E-10 mg/cell, respectively) and maltose (2.83E-10 and 8.96E-10 mg/cell, respectively), whereas they consumed onefold more glucose and maltose than their consumption in pure cultures during co-culture (**Figures 2E,F**). This, in turn, resulted in a twofold increase in acetate and lactate production in co-culture compared to the situation in mono-associations (**Figures 2A–C**). Although PRL2010 did not produce significant biomass from xylan or starch fermentation, it appears that glucose and maltose from degradation of starch was depleted from the growth medium regardless. This may be explained by the fact that PRL2010 is utilizing free glucose and/or maltose that may be present in very low amounts in the starch-supplemented growth medium (carbohydrate contaminants), thereby allowing very limited growth (**Figures 2E,F**). In general, organic acid production, and sugar consumption did not exhibit a linear correlation among the tested strains. This is likely due to the hydrolysis of dietary oligosaccharides yielding increased concentrations of monomeric and dimeric sugars derived from xylan or starch before they enter the bifid shunt (Ze et al., 2012).

However, since we are measuring the initial and final metabolites produced, we may not have detected intermediate liberated/produced metabolites from the provided carbon sources. Thus, the consumption kinetics is unclear and we cannot exclude the possibility that metabolite concentrations deviate over time due to glycosyl hydrolase activity.

### Transcriptomics of the Cross-feeding Features

**FIGURE 3:**
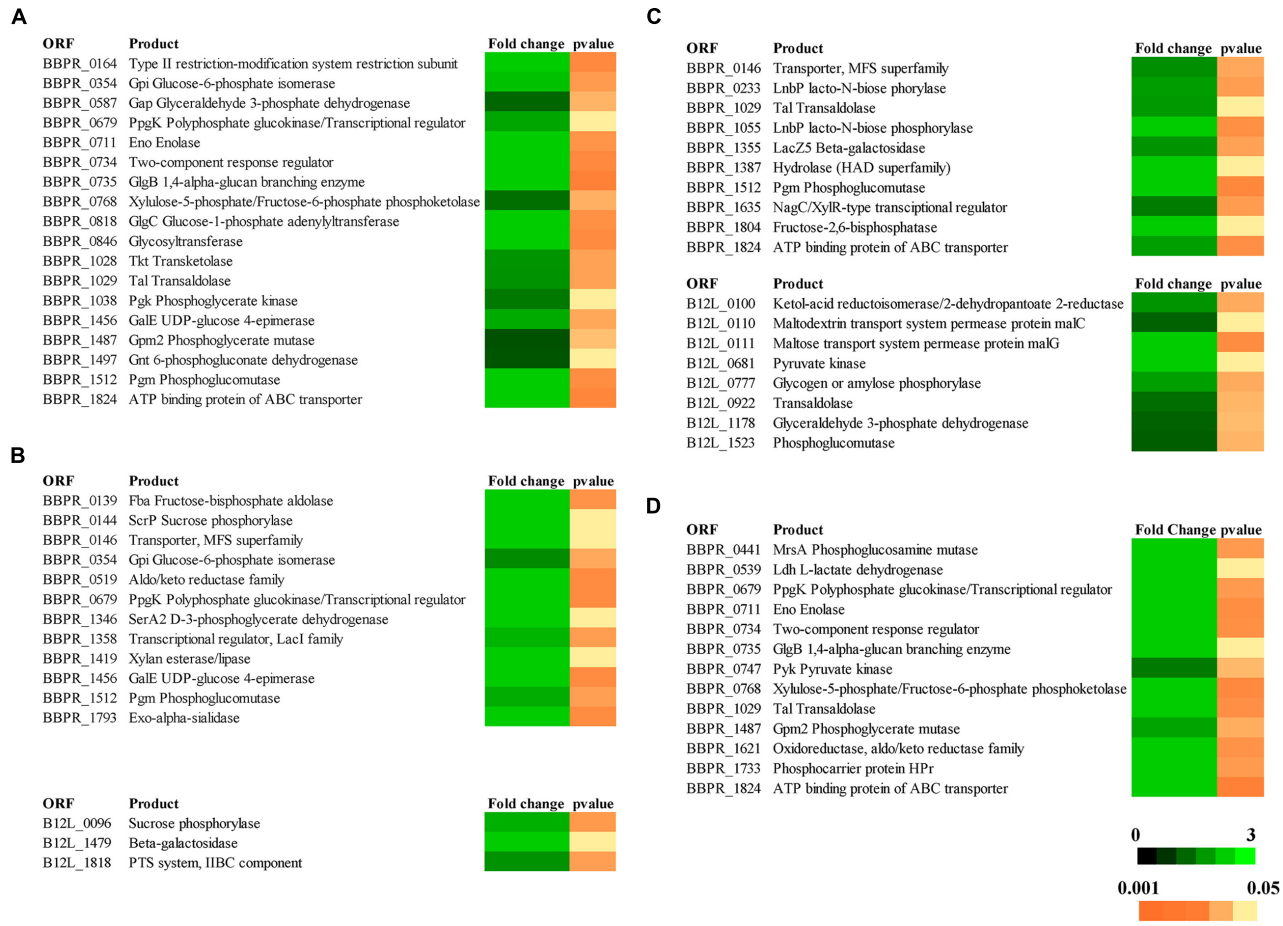
**Transcriptomic profiling of genes predicted to be involved in the metabolism of carbohydrates by bifidobacteria in response to the different bi- associations. (A)** The heat-map of transcriptional profiling of genes up-regulated in *B. bifidum* PRL2010 when this strain was co-cultivated with *B. adolescentis* 22L on starch-based medium. **(B)** The heat-map representing the transcriptional profiling of genes up-regulated in *B. bifidum* PRL2010 and in *B. breve* 12L when they were grown in bi-association on starch-based medium. **(C)** The heat-map representing the transcriptional profiling of genes up-regulated in *B. bifidum* PRL2010 and *B. breve* 12L when they were co-cultivated on xylan-based medium. **(D)** The heat-map representing the transcriptional profiling of the up-regulated genes in *B. bifidum* PRL2010 when was co-cultivated with *B. thermophilum* JCM1207 on xylan-based medium. Colors (black to green) represent the average signal intensity.

In order to evaluate the molecular aspects behind the cross-feeding activity as observed for some of the bi-associations involving the PRL2010–22L strain combination, or the PRL2010–12L strain combination when cultivated on starch, or the PRL2010–JCM1207 combination when grown on xylan, RNAseq experiments of these strain combinations cultivated on either of these substrates were performed. In order to increase the robustness of our RNAseq data, two technical replicates starting from the same library for each RNAseq trial were performed. When compared to the reference condition (mono-association) it was identified that the number of genes whose expression in either PRL2010 or the other strains, was significantly up-regulated (greater than or equal to twofold change, *p* < 0.005) ranged from 0 to 121. We used cluster orthologous gene (COG) analysis in order to identify differentially transcribed genes that may contribute to specific biological functions within the gut. As illustrated in **Figure 1**, carbohydrate metabolism, corresponding to COG category [G], is one of the COG functions of PRL2010 most significantly affected by the interaction with another bifidobacterial strain. This is probably due to a response to the presence of specific breakdown capabilities exploited by 22L, 12L, or JCM1207 cells. In this context, we observed an up-regulation of an ABC-type transporter-encoding gene (BBPR_1824), as well as an major facilitator superfamily (MFS) transporter-encoding gene (BBPR_0146), thus possibly involved in the uptake of simple sugars when co-cultivated with 22L or 12L cells on MRS containing starch as a unique carbon source (**Figure 3**). This finding can be explained by the fact that the extracellular amylases encoded by strains 22L (Duranti et al., 2014) and 12L (Bottacini et al., 2014) generate simple carbohydrates, which may then be imported by PRL2010 cells through its carbohydrate transporter arsenal (Turroni et al., 2012). Other transcriptionally induced genes of *B. bifidum* PRL2010 cells encompass enolase (BBPR_0711), glucose-6-phosphate isomerase (BBPR_354), phosphoglycerate kinase (BBPR_1038), glyceraldehyde 3-phosphate dehydrogenase (BBPR_0587), and phosphoglycerate mutase (BBPR_1487), whose functions are predicted to be related to (carbohydrate-dependent) energy production and conversion through the glycolytic pathway (Supplementary Figure S1). These observations indicate that PRL2010 cells have enhanced flux through their central fermentative pathway in the presence of 22L or 12L cells. Notably, we also observed the enhanced transcription of genes encoding the various subunits of the ATPase system of PRL2010 strain, which may occur in response to medium acidification as a result of enhanced metabolic activity. Co-cultivation of PRL2010 cells with strain JCM1207 on a xylan-based medium also increased the transcription levels of genes that are predicted to be involved in the carbohydrate metabolism (**Figure 3**). In particular, 121 genes of PRL2010 exhibited transcriptional up-regulation. The upregulated genes include several ORFs-encoding glycolytic enzymatic repertoire of PRL2010, such as enolase (BBPR_0711), phosphoglycerate mutase (BBPR_1487), and pyruvate kinase (BBPR_0747; **Figure 3**).

Interestingly, strain 12L displayed the transcriptional up-regulation of 21 genes when it was co-cultivated with PRL2010 on a starch-based medium. Notably, among the up-regulated genes, we observed B12L_1818 encoding a putative IIBC component of a phosphotransferase system (PTS), which is involved in carbohydrate metabolism (**Figure 3**). Co-cultivation of strain 12L with PRL2010 caused transcriptional up-regulation of 42 genes of *B. breve* 12L on xylan-based medium, some of which are known to be involved in energy generation through the bif shunt, such as pyruvate kinase (B12L_0681) and phosphoglucomutase (B12L_1523; **Figure 3**). This is consistent with the observation that 12L produces significantly more organic acid endproducts in axenic culture than when co-cultured with another bifidobacterial species. In contrast, the transcriptomes of strain 22L when cultivated on starch, and that of JCM1207 strain when grown on xylan-based medium were shown to be unaltered when these strains were co-cultivated with PRL2010 cells compared to transcriptome data obtained when these strains were in mono-association. Such findings confirmed the results achieved by growth experiments (see above).

## Conclusion

In this study, we assessed possible glycan cross-feeding activities between simple bifidobacterial communities when metabolizing complex carbohydrates that, being present in the diet, are expected to be commonly found in the human gut. Our results highlight the existence of a gut commensal relationship between different bifidobacterial species (Supplementary Table S1). We showed the *in vitro* ability of *B. bifidum* PRL2010 to cross-feed on sugars released by the starch- and/or xylan-degrading activities of *B. adolescentis* 22L, *B. breve* 12L, and *B. thermophilum* JCM1207. The generated information advances our knowledge on the metabolic adaptability and versatility of these strains, which no doubt will facilitate colonization of the human gut. Interestingly, when *B. bifidum* PRL2010 was co-cultivated with *B. breve* 12L we observed effects on the transcriptomes of both strains, apparently affecting the glycolytic pathway. The observed transcriptional changes may represent a molecular example of a mutualistic relationship between these two bifidobacterial strains, perhaps being a reflection of their common ecological origin, i.e., the infant gut.

The precise characterization of such complex interactions between gut microbiota members is pivotal in the process of modulation of the composition of the intestinal microbial population, especially during probiotic treatments.

## Conflict of Interest Statement

The authors declare that the research was conducted in the absence of any commercial or financial relationships that could be construed as a potential conflict of interest.

## Footnotes

1 http://code.google.com/p/ea-utils

2 http://www-huber.embl.de/users/anders/HTSeq/doc/overview.html

## Supplementary Material

FIGURE S1 **Evaluation of the cell density of bifidobacterial strains by qRT-PCR assays**. Evaluation of the cell density of mono- and co-cultivation of *Bifidobacterium breve* 12L, *Bifidobacterium adolescentis* 22L, *Bifidobacterium bifidum* PRL2010, and *Bifidobacterium thermophilum* JCM1207 strains on xylan- and starch-based medium by qRT-PCR experiments. *y*-axis represents the genome copy number/ml of bacterial culture, *x*-axis showed the name of the strains involved in mono- and bi-associations. Empty bars represent the amount of cells present at T0. Asterisks indicate that the presented data display a significant deviation, i.e., **p* < 0.05 or ***p* < 0.01, with respect to the data obtained for the mono-association.
