# Supplementary figures and images for: Glycan cross-feeding activities between bifidobacteria under in vitro conditions
Source: Front Microbiol. 2015 Sep 24;6:1030. doi: 10.3389/fmicb.2015.01030 (PMC4585166; doi:10.3389/fmicb.2015.01030)

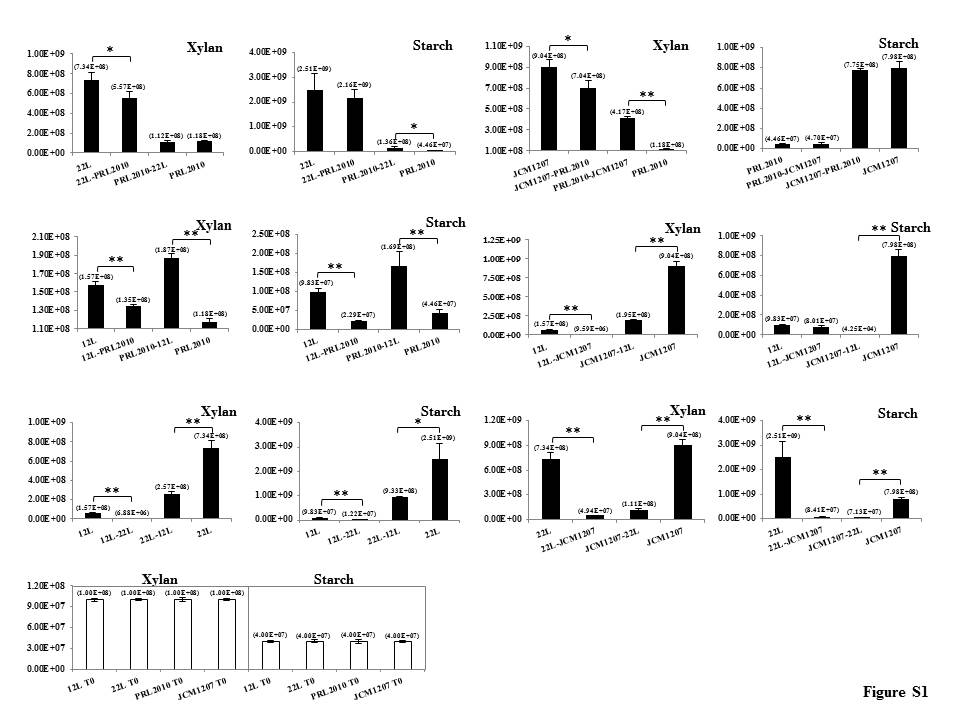

Supplement: FIGURE S1 — Evaluation of the cell density of bifidobacterial strains by qRT-PCR assays. Evaluation of the cell density of mono- and co-cultivation of Bifidobacterium breve 12L, Bifidobacterium adolescentis 22L, Bifidobacterium bifidum PRL2010, and Bifidobacterium thermophilum JCM1207 strains on xylan- and starch-based medium by qRT-PCR experiments. y-axis represents the genome copy number/ml of bacterial culture, x-axis showed the name of the strains involved in mono- and bi-associations. Empty bars represent the amount of cells present at T0. Asterisks indicate that the presented data display a significant deviation, i.e., *p < 0.05 or **p < 0.01, with respect to the data obtained for the mono-association. [file Image_1.JPEG]
