# Supplementary material for: Glycan cross-feeding activities between bifidobacteria under in vitro conditions
Source: Front Microbiol. 2015 Sep 24;6:1030. doi: 10.3389/fmicb.2015.01030 (PMC4585166; doi:10.3389/fmicb.2015.01030)
Supplement: Supplementary file 2 [file Table_1.DOCX]

| **starch** | PRL2010 | 22L | 12L | JCM1207 |  |  |  | **xylan** | PRL2010 | 22L | 12L | JCM1207 |
| --- | --- | --- | --- | --- | --- | --- | --- | --- | --- | --- | --- | --- |
| PRL2010 | **-** | **- +** | **+ +** | **+** |  |  |  | PRL2010 | **-** | **-** | **+ +** | **+ +** |
| 22L | **+ +** | **+** | **-** | **- +** |  |  |  | 22L | **+** | **+** | **-** | **-** |
| 12L | **- -** | **-** | **+** | **+** |  |  |  | 12L | **+ -** | **-** | **+ +** | **-** |
| JCM1207 | **-** | **- +** | **-** | **+** |  |  |  | JCM1207 | **+ -** | **-** | **-** | **+** |

**Table S1.** Schematic representation showing interactions of PRL2010, 12L, 22L and JCM1207 strains in mono-association and bi-association.

Blue symbols: growth data

Red symbols: transcriptomic data

Green symbols: metabolic data
